# Mother–Child Attachment Relationship in Pregnancy, Postpartum, and Early Childhood: Current Status and New Research Perspectives

**DOI:** 10.3390/ejihpe14080148

**Published:** 2024-08-01

**Authors:** Valentina Lucia La Rosa, Elena Commodari

**Affiliations:** Department of Educational Sciences, University of Catania, Via Biblioteca 4, 95124 Catania, Italy; e.commodari@unict.it

The mother–child attachment relationship is a cornerstone of human development, with profound implications for the well-being of both the mother and child. Attachment theory, pioneered by John Bowlby [1,2] and Mary Ainsworth [3,4], has long recognized the critical role of the mother–child bond in shaping a child’s emotional, social, and cognitive development. Secure attachment, characterized by a child’s trust in the mother’s availability and responsiveness, provides a foundation for healthy development and resilience [5]. Insecure attachment, on the other hand, can lead to a variety of emotional and behavioral difficulties in children, including anxiety, depression, and aggression [6,7]. The quality of the mother–child attachment relationship is influenced by a variety of factors, including maternal sensitivity and responsiveness, infant temperament, and environmental stressors [8,9]. Understanding these factors and how they interact is critical for promoting secure attachment and reducing the risk of adverse outcomes across the lifespan [10,11,12,13].

In recent years, there has been a growing recognition of the importance of examining the mother–child attachment relationship from a multifaceted perspective, including not only the traditional focus on the postpartum period, but also the prenatal and early childhood periods, as they are formative in establishing and shaping attachment bonds [14]. This broader perspective is essential because the foundations of attachment are laid during pregnancy, with maternal psychological well-being and expectations significantly influencing the early stages of the mother–child bond [15,16,17]. The quality of this relationship continues to evolve throughout early childhood, with parenting practices and children’s developing emotion regulation playing a critical role in shaping attachment security [18,19].

This Special Issue entitled “Mother–Child Attachment Relationship in Pregnancy, Postpartum, and Early Childhood: Current Status and New Research Perspectives” delves into the intricate dynamics of this bond, exploring its manifestations, predictors, and consequences at various stages of early life. The articles in this Special Issue cover a wide range of topics that span the trajectory of early development and provide a rich overview of research findings, theoretical insights, and clinical implications that collectively contribute to a deeper understanding of the complexities of the mother–child attachment relationship.

A notable theme that emerges from this collection is the importance of maternal mental health in shaping the quality of the mother–child attachment relationship. Leong et al. [20] examined the impact of early parenting interactions on first-time mothers’ postnatal depression and maternal competence, using recently revised and newly developed schema therapy-informed tools. Their findings underscore the lasting effects of both positive and negative early interactions and highlight the importance of fostering a nurturing environment to support maternal mental health and enhance mother–child bonds. Similarly, Estlein and Shai [21] examined the predictive role of dyadic prenatal co-parenting behaviors on postpartum depressive symptoms in first-time parents. Their longitudinal study highlighted the importance of addressing co-parenting dynamics during pregnancy as a potential avenue for reducing the risk of postpartum depression. These studies are consistent with a growing body of evidence suggesting that maternal mental health, particularly depression and anxiety, can significantly impact the quality of the mother–child attachment relationship [22,23].

Another key theme is the need to explore new psychometric tools for the postpartum period, as traditional measures of psychological variables often fail to capture the nuanced experiences of postpartum women [24,25,26]. In this regard, Ionio et al. [27] investigated the relationship between specific postpartum anxiety and maternal-infant bonding among Italian women using the Italian version of a new instrument, the Postpartum Specific Anxiety Scale (PSAS-IT), which is specifically designed to measure postpartum anxiety [28,29]. Their study found an association between specific postpartum anxiety symptoms and attachment problems, highlighting the need for early identification and interventions to promote healthy attachment. This study contributes to a deeper understanding of how specific anxiety, beyond generalized anxiety, may uniquely affect the mother–infant bond during the postpartum period and provides robust evidence for the predictive validity of the PSAS-IT in assessing anxiety problems among Italian women in the postpartum period.

The articles in this Special Issue also shed light on the complex interplay among maternal self-efficacy, breastfeeding practices, and infant health. Brani et al. [30] conducted a longitudinal study in Greece to examine the association between maternal breastfeeding self-efficacy and breastfeeding outcomes in both high-risk and normal-risk pregnancies. Their findings highlight the critical role of maternal self-efficacy in breastfeeding success, which is influenced by individual psychological factors and broader socio-cultural contexts. This research is consistent with previous studies that have highlighted the importance of maternal self-efficacy in promoting breastfeeding initiation and duration [31,32,33].

In addition to maternal factors, this Special Issue considers the child’s perspective on attachment. Augustin et al. [34] examined the role of child attachment and maternal emotional availability in predicting ADHD symptoms in middle childhood, suggesting that maternal emotional availability, particularly non-hostility, is associated with lower ADHD symptoms, independent of child attachment representations. These findings add to the growing body of evidence suggesting that maternal emotional availability and attachment style play a critical role in children’s emotional and behavioral development [35,36,37,38]. Rolo et al. [39] examined the mediating role of children’s emotion regulation in the relationship between mothers’ responses to children’s emotions and children’s behavior in school. Their findings suggest that mothers’ responses to children’s emotions may significantly influence children’s classroom behavior through mechanisms involving children’s emotion regulation, and highlight the importance of considering children’s emotion regulation as a potential mediator in the relationship between maternal behaviors and child outcomes [40].

Collectively, the articles in this Special Issue provide a comprehensive and nuanced understanding of the mother–child attachment relationship and highlight the importance of considering multiple factors, including maternal mental health, anxiety, self-efficacy, parenting practices, and child emotion regulation, in understanding the complexity of this bond.

While this Special Issue has made significant strides in advancing our knowledge of the mother–child attachment relationship, several avenues for future research warrant consideration. First, there is a need for more culturally diverse studies to understand how cultural norms and values shape attachment behaviors and outcomes. Research should examine how cultural differences in parenting practices and beliefs about child development influence the mother–child attachment relationship. Second, longitudinal research is essential for examining the long-term effects of early attachment experiences on later developmental outcomes. Studies should follow mother–child dyads over time to assess how attachment patterns evolve and influence children’s social, emotional, and cognitive development across developmental stages. Third, more intervention studies are needed to develop and evaluate effective interventions to promote secure attachment and mitigate the negative effects of insecure attachment. Research should also focus on developing and testing interventions that target specific risk factors for insecure attachment, such as maternal mental health problems, parenting stress, and adverse childhood experiences. Finally, future research should explore the role of fathers and other caregivers in attachment relationships, as well as the impact of technology and social media on attachment behaviors.

In conclusion, this Special Issue is a valuable resource for researchers, clinicians, and policymakers interested in the mother–child attachment relationship. The articles in this Issue offer new insights into the complexity of this bond and highlight the importance of considering a multifaceted approach for understanding and promoting healthy attachment relationships. Building on the knowledge presented in this Special Issue, we can continue to advance our understanding of this critical aspect of human development and promote optimal outcomes for mothers and children.

## Data Availability

Data sharing is not applicable to this article.
